# Beyond the Complement Cascade: Insights into Systemic Immunosenescence and Inflammaging in Age-Related Macular Degeneration and Current Barriers to Treatment

**DOI:** 10.3390/cells12131708

**Published:** 2023-06-23

**Authors:** Adnan H. Khan, Itay Chowers, Andrew J. Lotery

**Affiliations:** 1 Clinical and Experimental Sciences, Faculty of Medicine, University of Southampton, Southampton SO17 1BJ, UK; 2 Southampton Eye Unit, University Hospital Southampton NHS Foundation Trust, Southampton SO16 6YD, UK; 3 Department of Ophthalmology, Hadassah-Hebrew University Medical Center, Jerusalem 91120, Israel; 4 Faculty of Medicine, The Hebrew University of Jerusalem, Jerusalem 91121, Israel

**Keywords:** immunosenescence, inflammaging, age-related macular degeneration, macular degeneration, senolytics

## Abstract

Landmark genetic studies have revealed the effect of complement biology and its regulation on the pathogenesis of age-related macular degeneration (AMD). Limited phase 3 clinical trial data showing a benefit of complement inhibition in AMD raises the prospect of more complex mediators at play. Substantial evidence supports the role of para-inflammation in maintaining homeostasis in the retina and choroid. With increasing age, a decline in immune system regulation, known as immunosenescence, has been shown to alter the equilibrium maintained by para-inflammation. The altered equilibrium results in chronic, sterile inflammation with aging, termed ‘inflammaging’, including in the retina and choroid. The chronic inflammatory state in AMD is complex, with contributions from cells of the innate and adaptive branches of the immune system, sometimes with overlapping features, and the interaction of their secretory products with retinal cells such as microglia and retinal pigment epithelium (RPE), extracellular matrix and choroidal vascular endothelial cells. In this review, the chronic inflammatory state in AMD will be explored by immune cell type, with a discussion of factors that will need to be overcome in the development of curative therapies.

## 1. Introduction—Age-Related Macular Degeneration (AMD) and the Complement System

Age-related macular degeneration (AMD), a progressive retinal disease that results in the loss of central vision, is predicted to affect 288 million people worldwide by 2040 [1]. Dry or atrophic AMD (aAMD) is associated with progressive degeneration of RPE cells and choroid. This leads to secondary photoreceptor damage and eventually, the clinical phenotype of geographic atrophy (GA) [2,3]. Wet or neovascular AMD (nAMD) is a result of macular neovascularization (MNV), resulting in rapid vision loss [2,3]. The chronic course of aAMD means that patients might encounter significant reduction in or loss of central vision. Similarly, in the long-term, nAMD patients, despite the application of available anti-vascular endothelial growth factor (VEGF) therapies, show a mean reduction in visual acuity [4].

In the healthy eye, the retinal pigment epithelium (RPE) is a monolayer of pigmented cells that provides structural and metabolic support to the retinal photoreceptors [2,3]. The choroid, underlying the RPE, is a heterogenous tissue that provides significant support to the retina (including ~85% of the blood to the outer retina) and consists of several different cell populations [2,3,5]. This includes a wide array of leukocytes [5]. The earliest clinical sign of AMD is the formation of macular drusen (visible as yellow spots on fundus images) at the sub-RPE level and, occasionally, RPE pigmentary abnormalities [6].

AMD has a multifactorial etiology, including diet, smoking, photo-oxidative and hypoxic stress [7,8,9,10]. It is well-known that the rate of AMD progression varies in patients, with some patients being fast progressors: at least 10% develop MNV [1]. Doppler studies from AMD patients have revealed decreased choroidal blood flow during the progression of AMD, with the greatest impairment of flow observed in eyes with the highest risk of developing choroidal neovascularization [11,12,13,14,15]. Vascular dropout has been shown to precede neovascularization in human eyes. In addition, decreased choriocapillaris vasculature has been associated with an increasing cross-sectional area of drusen and basal deposits [16].

There is a significant evidence base for a genetic component in AMD, and numerous single nucleotide polymorphisms (SNPs) have been associated with a patient’s risk of developing AMD [17]. The concept of complement activation having a major role in AMD pathogenesis is supported by genetic studies demonstrating that mutations in a number of complement regulatory genes are strongly associated with all stages of AMD [10,18]. The activation of either the classical pathway (initiated by binding of C1q to immune complexes) or alternative pathway (the association of C3b and properdin, mannose-binding lectin, or microorganism-associated molecular patterns (MAMPs)) of complement activation results in the formation of convertases that cleave the C3 and C5 proteins [19]. Cleavage of C3 and C5 proteins leads to the subsequent formation of the membrane attack complex (MAC; C5b-9) and generation of active fragments. The insertion of MAC into cell membranes results in cytolysis or cell activation [19] and complement activation is associated with drusen formation in AMD patients [20]. Uncontrolled activation of the complement pathway is limited by a set of complement regulatory proteins, including factor H and factor I, which regulate the alternative pathway [21]. The presence of very rare coding variants (frequency < 0.1%) in the complement factor H (*CFH*) and complement factor I (*CFI*) genes have been associated with AMD pathogenesis [17]. Genes independent of the complement pathway have also been implicated in AMD pathogenesis, including genes regulating lipid metabolism, and oxidative stress, such as the age-related maculopathy susceptibility 2 (*ARMS2)* [22] and TIMP metallopeptidase inhibitor 3 (*TIMP3*) genes [17].

Current treatments available to reduce the risk of progression to late-stage AMD include dietary vitamins [23]. One large, randomized phase 3 clinical trial showed no benefit of complement inhibition to reduce the rate of geographic atrophy growth [24]. A more recent phase 2 clinical trial of the C3 inhibitor pegcetacoplan in GA (the DERBY and OAKS studies) showed modest reductions in the rate of growth of lesions, although this included cases of conversion to nAMD [25]. At the time of publication, pegcetacoplan has been approved by the FDA [26], is under review by the regulatory authorities in Europe, and phase 3 clinical trial data have not yet been published. A phase 2/3 clinical trial of the C5 inhibitor avacincaptad pegol in GA (the GATHER1 study) also showed a modest reduction in GA lesion growth over a 12-month period [27]. No other phase 3 clinical trials have shown a clear benefit of complement inhibition in AMD.

Genetic variation in complement regulatory genes do not fully account for disease development [28]. In this review, we will discuss rapidly growing evidence suggesting that factors beyond the complement cascade contribute to AMD pathogenesis, including the negative impact of an aging immune system and a chronic inflammatory state (both systemic and local). We will highlight specific components of innate and adaptive immunity (cell types) with a discussion of factors that will need to be overcome in the development of curative therapies.

## 2. Parainflammation and Immunosenescence

In the aging eye, and predominantly at the level of the RPE, there is an accumulation of reactive oxygen species (ROS), lipofuscin and other by-products of photoreceptor renewal such as A2E [29]. These by-products alter the metabolism and health of the RPE. The state of ‘para-inflammation’ has been described as maintaining tissue homeostasis [30]. It has features between basal and inflammatory states, resulting in low-grade inflammatory-based clearance of noxious stimuli [31].

There is increasing evidence, which we will describe below, that the dysregulation of the immune response causes AMD-like phenotypes. With increasing age, there is a general decline in the immune system and thymic function, termed immunosenescence. Immunosenescence has increasingly been implicated in driving systemic ageing [32]. It has been speculated that immunosenescence alters the equilibrium maintained by para-inflammation, with a loss of systemic control of many aspects of the immune system and the release of new inflammatory factors, leading to increased leukocyte infiltration and activation at sites of tissue damage [31]. Therefore, immunosenescence has a negative impact on a wide range of diseases.

Immunosenescence is thought to result from the accumulation of senescent cells within both the innate and adaptive branches of the immune system [33,34]. Furthermore, there is increasing evidence of a convergence of innate and adaptive features during immunosenescence, with acquisition of innate-like functions by T cells, such as terminal differentiation, rapid effector function, and a functional shift away from antigen specificity [34]. Migration of senescent cells and their secretions (in the form of pro-inflammatory cytokines such as IL-6 in the blood) can sustain senescence locally and spread its effects systemically [34].

At the cellular level of senescence, there is irreversible cell cycle arrest (G1 arrest) in both mitotic [35,36] and an increasing number of post-mitotic cells [37]. Established biomarkers of senescent cells include proteins that result from the activation of tumor suppressor pathways (e.g., the p53 tumor suppressor or the cyclin-dependent kinase inhibitor p16^INK4A^) [34,38], p38, MAPK, p21^CIP1^, RB and cyclin-dependent kinases (CDKs) [39]. Cellular senescence is believed to be the response to intrinsic signals such as telomere shortening and metabolic dysfunction, in addition to external cues such as oxidative stress [40].

Senescent cells also display functional and morphological differences, including increased activity of senescence-associated β-galactosidase (SA-β-Gal) and the accumulation of foci containing DNA damage, e.g., double-stranded breaks, modifications to chromatin and altered mitochondrial metabolism [39,40,41]. With immunosenescence, these morphological and functional differences may cause a loss of immune cell effector function, leading to over-production of pro-inflammatory factors including cytokines, growth factors, proteases and lipids [collectively termed the senescence-associated secretory phenotype (SASP)] [39,42] and a chronic inflammatory state [34]. Senescent changes have been observed in retinal tissue in AMD, and they may exert cytotoxic/neovascular effects via SASP [40]. SASP has been associated with AMD because a number of pro-inflammatory cytokines have been detected systemically (in the serum) or locally (in the aqueous humor) of AMD patients, including IL-6, IL-8, IL-12, MCP-1, TNF-α, IL-1α, IL-1β, and IL-17 [43,44].

There is evidence of notable similarities between immunosenescence and cellular senescence in immune cells, particularly macrophages and lymphocytes [33,40]. These similarities include the senescence markers described above, such as telomere shortening, the pro-inflammatory mediators of SASP, p16^INK4A^, p21^CIP1^, and SA-β-Gal [33,45,46]. Interestingly, retinal drusen in patients with myeloproliferative blood cancers are associated with an increased proportion of senescent T cells [47]. Furthermore, it has been suggested that in chronic myeloid leukemia, the immunological phenotype may accelerate drusen formation [47].

It is well-known that immunosenescence is associated with a reduction in tolerance induction mechanisms and an increase in the levels of autoantibodies (both systemically and locally) [48,49]. Whether this is a cause or result of AMD is yet to be determined and the evidence is discussed below.

Importantly, it has been demonstrated that clearance of senescent cells can delay onset and reduce severity of several age-associated diseases (including ocular disease) in animal models [50]. This supports the concept of “senolytic therapy” to improve the elimination of senescent cells whilst boosting immune function [51].

## 3. Inflammaging and the Chronic Inflammatory State in AMD

Inflammation constitutes a significant element of the ageing process, hence the term “inflammaging”. This is characterized by a low-grade, chronic inflammatory state and a shift to increased tissue expression of pro-inflammatory cytokine and chemokines [52]. A summary of evidence for immunosenescence and inflammation with aging (both systemic and local), by immune cell type, is presented in Table 1.

This chronic inflammatory state has relevance to, and implications for, AMD progression. For example, whereas drusen in AMD are defined anatomically as sub-RPE deposits, reticular pseudodrusen (RPD) are sub-retinal drusenoid deposits that are located internal to the RPE [53]. The presence of RPD confers a 4-8 fold greater risk of 5-year progression to the advanced stages of AMD, including geographic atrophy [54]. Sivaprasad and colleagues have speculated that RPD may be a manifestation of the failure to regulate age-associated RPE damage via para-inflammation in addition to heightened immune-mediated responses contributing to RPE and retinal damage [55]. There is also evidence of chronic inflammatory diseases (including those associated with infection) being associated with AMD progression: The 15-year follow-up examination of the Beaver Dam Eye Study, which investigated pulmonary disease and AMD, demonstrated that independently of smoking, a history of emphysema and respiratory symptoms is associated with the incidence and progression of AMD [56].

Under physiological conditions, the RPE normally promotes T cell unresponsiveness (anergy) [57] and/or actively eliminates infiltrating T cells via Fas-mediated apoptosis [58]. However, the immune suppressive functions of the RPE can be compromised. This is associated with complement activation [28], induction of proinflammatory cytokines, and activation of both innate and adaptive immune responses [30,31]. MHC class II expression has been demonstrated on RPE with associated activation of recruited T cells [59].

Retinal microglia are tissue-resident macrophages involved in local immune surveillance and tissue repair [60]. Similar to RPE cells, and under physiological conditions, retinal microglia express low levels of MHC class II [61]. Therefore, retinal microglia are immunoregulatory in nature under physiological conditions, with a low potential for T cell activation [61,62]. Senescent microglia contribute to a chronic inflammatory state and loss of tissue homeostasis in AMD [63,64]. Microglia from aged murine retinae express changes in genes controlling inflammatory activity, including NF-κB, complement factor C3 and CFB, indicating a role of senescent microglia in promoting immune dysregulation [65]. It has been demonstrated using rodent models that, with aging and disease conditions, MHC class II expression is enhanced on retinal microglia, with these cells resembling dendritic cell populations [61,66]. The changes in retinal microglia surface MHC class II expression correlate with the structural changes in the RPE [67] and T cell infiltration into areas of RPE degeneration. These immunostimulatory microglia are capable of activating T cells and are necessary for the development of ocular autoimmunity [68]. Interestingly, in rodent models, aging is not only associated with microglial senescence but also with secondary neuroinflammation and worse neurological outcomes after traumatic brain injury [69]. Similarly, during aging and in the pathogenesis of AMD, accumulation of microglia in the sub-retinal space and inflammatory activation of retinal microglia can also result in disruption of the eye’s immune privilege and production of retinal autoantibodies [70].

**Table 1:** Summary of evidence for immunosenescence and inflammation with aging—systemic vs. local.

Immune Compartment or Tissue	Cell or Tissue Type	Systemic	Local (Choroid and Retina)
Innate Immunity	Neutrophils	Impaired trafficking (e.g., retrograde motility into vascular lumen from tissue) with aberrant pro-inflammatory cytokine production [71]. Reduced phagocytic activity and elevated production of pro-inflammatory mediators [72].	Reduced phagocytic activity and elevated production of pro-inflammatory mediators [72].
	Monocytes–Macrophages	Reduced phagocytic activity and elevated pro-inflammatory mediators [73]. Markers of macrophage senescence: SA-β-Gal, telomere shortening, p16^INK4A^, p21^CIP1^ [34,38,39].	Retinal microglia: elevated MHC Class II [61,66] and NF-κB expression [65]. Reduced phagocytic activity and elevated pro-inflammatory mediators.
	Mast cells	Increased numbers of mast cells in close apposition to venular walls [74,75]. CXCL1 production by mast cells and context-dependent SASP [74,75].	Numbers of mast cells relatively unaffected by age. Oxidative environment drives production of pro-inflammatory mediators by mast cells [74,75].
	Natural Killer (NK) cells	Inhibition of NK cell activity by senescent cells via expression of HLA-E [76]. Senescent cell evasion of NK cells via NKG2D expression [77]. Inconsistent evidence: Proportion of CD56^++^ NK cells and CD56^+^CD16^+^ NK cells with age.	Limited evidence of NK cell activity in the choroid/retina of aging eyes.
Adaptive Immunity	T cells	Reduction in peripheral T cell counts [29,31]. Increased frequencies of memory CD4^+^ and CD8^+^ T cells [29,31]. Loss of costimulatory molecules CD28, CD27 and CD40L [78,79]. Increased IFN-γ production associated with CD8^+^CD28^-^CD57^+^ T cell subpopulation [80]. Increased frequency of Th17 cells and reduced inducibility/stability of Tregs [81]. Markers of T cell senescence: SA-β-Gal, telomere shortening, p16^INK4A^, p21^CIP1^ [34,38,39].	T cell-specific genes upregulated in the retina and RPE/choroid with increasing age, including genes for T cell chemotaxis [82,83].
	B cells	Circulating B cell numbers generally decrease with age: decreased production of high-affinity protective antibodies	Limited evidence of B cell numbers or activity in donor eyes from aged patients.
Interface- “Professional” antigen presenting cells (APCs)	Dendritic cells (DCs)	No strong evidence to suggest change in DC numbers or activity with aging—phagocytosis of antigens appears unaffected, and there are contrasting data about cytokine production.	Population of DCs detected based on choroidal transcriptomes [84].
Stroma, Vasculature or Peripheral Blood	Tissue (as specified)	Visceral adipose tissue: increased pro-inflammatory macrophages; decreased M2-like immunoregulatory macrophages [85,86]. SPARC expression by adipose tissue [87]. Increased infiltration of myeloid cells in tissues. ISG and IRF7 expression by stromal tissue [88]. Elevated DARC (ACKR1) expression by aged venules [71].	RPE: elevated MHC Class II expression [59]. Reduced Fas expression [58]. Markers of senescent cells in the choroid/RPE/retina: p16^INK4A^, p53, p38, MAPK, p21^CIP1^, RB, cyclin-dependent kinases. Senescence of retinal vascular endothelial cells in aging retina [89]. Choriocapillaris: elevated ICAM-1; Reduced CD34 [90]. Elevated DARC (ACKR1) expression by aged venules [71].
	Serum or plasma	SASP markers (detected in serum): IL-6, IL-8, IL-12, MCP-1, TNF-α, IL-1α, IL-1β, and IL-17.	

Abbreviations: Duffy antigen receptor for chemokines (DARC); Intercellular adhesion molecule 1 (ICAM-1); Interferon-γ (IFN-γ); Interferon regulatory factor 7 (IRF7); Interferon-stimulated gene (ISG); Natural Killer group 2D (NKG2D); Senescence-associated β-galactosidase (SA-β-Gal); Senescence-associated secretory phenotype (SASP); Secreted protein acidic and rich in cysteine (SPARC).

The transcriptome of choroidal endothelial cells has been compared at the single-cell level in young and older patients [90]. This has identified several pro-inflammatory genes enriched in the aging choriocapillaris. For example, intercellular adhesion molecule 1 (ICAM-1) facilitates leukocyte–endothelial cell interactions and was enriched in the adult choriocapillaris [90]. In contrast, CD34, a highly glycosylated sialomucin, inhibits leukocyte extravasation through the vasculature and was enriched in infant choriocapillaris [90]. Both ICAM-1 and CD34 demonstrated qualitative expression differences at the protein level between infant and adult choriocapillaris as well.

Similar to the concept of senolytic therapy mentioned above, there is significant evidence that the mechanisms which drive chronic low-grade inflammation can be targeted to delay degenerative changes [91].

## 4. Immunosenescence and the Innate Immune System

Although there is significant evidence of immunosenescence affecting the adaptive immune system and CD8^+^ T cells in particular [29,33], changes have been observed in the innate immune system. The innate immune system has a major role in generating the inflammatory response, activating the complement cascade, maintaining the blood–retina barrier and activating the adaptive immune response via antigen presentation. Cell types of the innate immune system include monocytes and monocyte-derived (tissue-based) macrophages, neutrophils, and Natural Killer (NK) cells. With immunosenescence, these cell types acquire the hallmark features of cell senescence, including impaired function [72,92]. Aging innate immune cells such as macrophages and neutrophils display reduced phagocytic activity and express increased levels of pro-inflammatory mediators. Together, this promotes local and systemic inflammaging and enables the survival of senescent cells that are usually cleared by resident immune cells such as microglia [31,40,93]. The role of the innate immune system in AMD can be demonstrated by elevated plasma levels of activated complement factor 3 (C3a) [94] and C-Reactive Protein (CRP), an acute phase reactant and marker of inflammation [95,96], in AMD patients relative to age-matched controls.

The inflammasome is a protein complex associated with the innate immune system and consists of a NOD-like family member (e.g., NLRP3), the adaptor protein ASC, and the caspase-1 protein. The inflammasome is activated rapidly by danger signals, culminating in the maturation of IL-1β and IL-18, which can be followed by necroptosis or apoptosis [29]. Under physiological conditions, the retina and RPE produce natural mediators (e.g., resolvins) to suppress inflammasome-mediated inflammation, and inflammasome activations is downregulated by autophagy [97]. The components of drusen, such as the complement component C1q, have been shown to activate the inflammasome [98]. Furthermore, inflammasome activation has been implicated in the pathogenesis of AMD [29]. Elevated expression of NLRP3, IL-18 and activated caspase-1 (all evidence of inflammasome activation) has been detected in the RPE of eyes from AMD patients [99]. It is suggested that the retina can respond to AMD-specific danger signals by activation of the inflammasome [29].

A summary of immunosenescence and inflammation in AMD (both systemic and local), by immune cell type, is presented in Table 2.

### 4.1. Macrophages and Monocytes Contributing to Systemic and Local Immunosenescence and Inflammaging

Voigt et al. recently identified clusters of mononuclear phagocytes based on their choroidal transcriptomes [100]. Resident macrophages were distinguished from infiltrating monocyte-derived ‘inflammatory’ macrophages based on characteristic gene expression of these populations. Resident macrophages proliferate locally within the choroidal tissue, recruit monocyte-derived inflammatory macrophages, and contribute to tissue homeostasis by phagocytosing cellular debris and resolving inflammation [100]. In comparison, bone marrow-derived monocytes recruited to the choroid (thereafter converting to infiltrating macrophages) can drive inflammation by ROS and cytokine production [100]. Both circulating or infiltrating monocytes/macrophages associated with AMD, in addition to senescent retinal microglia, have been demonstrated to express SASP, including the inflammatory factors IL-1α, IL1-β, IL-6, IL-8, IL-12, TNF-α, C3, CFB, CXCL1, TGF-β and nitric oxide [43,65]. Macrophages are generally characterized in vitro as pro-inflammatory (M1) or immunoregulatory-promoting vascular stability (M2). However, in vivo, they exist more on a continuous spectrum [84,101].

Ageing is linked to increased visceral adiposity, which is, in turn, associated with chronic inflammation [102]. ‘Anti-inflammatory’ macrophages are decreased in adipose tissue with ageing and obesity, whilst pro-inflammatory macrophages are increased [85,86]. Furthermore, macrophage activation has been shown to drive sterile inflammation in aging and obesity [103]. The association of senescence with impaired macrophage function has recently been demonstrated by our group: senescent macrophages promote AMD via impaired cholesterol efflux [104]. In addition, Lin et al. showed that senescent macrophages induce the production of atypical lipid species within the retina via miRNA-regulated gene transcription, resulting in aberrant lipid metabolism and inflammation [105].

The secreted protein acidic and rich in cysteine (SPARC) is a matricellular protein that is expressed constitutively in several tissues, including adipose tissue [87]. However, elevated expression of SPARC is associated with chronic inflammation and fibrosis, and systemic levels regulate inflammation through interaction with cell surface matrix-associated molecules [106,107,108]. SPARC expression changes according to weight gain or loss in obese individuals [109] and stimulates proinflammatory gene expression in macrophages [108,110]. Ryu and colleagues recently demonstrated that increased SPARC concentration reversed established M2-like anti-inflammatory macrophages (CD11c+CD206+) into an M1-like (CD11c+CD206-) pro-inflammatory state [88].

Inflammaging is associated with increased interferon-stimulated gene (ISG) expression, and elevated SPARC levels induced by ISG expression, itself dependent on upregulation of transcription factor IRF7 [88]. Furthermore, adipocyte-specific reduction in SPARC reduced ISG expression, which protected against adipocyte inflammation and inflammaging [88]. SPARC not only regulates the ECM, but also has a role in polarizing macrophages for tissue homeostasis. The polarization to M1-type/pro-inflammatory macrophages is required to produce ECM modulating factors such as matrix metalloproteinases (MMPs) [111] and pro-inflammatory cytokines such as IL-1β, TNF-α, IL-6, and GM-CSF. SPARC-deficient mice are protected from age-related inflammation with associated reduction in macrophage infiltration and pro-inflammatory cytokine expression [108].

The infiltration of microglia and macrophages to sites of retinal injury can promote the growth of neovascular lesions [112,113]. Both macrophages and RPE cells are a major source of pro-angiogenic factors such as VEGF-A [114]. As stated above, resident macrophages and dendritic cells are normally found in the choroid. However, with aging and during the breakdown of the blood–retina barrier, they are recruited from the choroid or the circulation into the retina where they can promote disease [29].

Our group has established protocols to derive macrophages from the monocytes of patients with AMD [115]. High expression levels of IL-6 (a potent pro-inflammatory cytokine associated with SASP), G-CSF, MIP-3a and MCP-3 can be detected in M1 macrophage-cultured media relative to M2 macrophages [115]. We have demonstrated that adoptively transferred human macrophages from AMD patients (via intravitreal injection) are pro-angiogenic, promoting CNV relative to macrophages from control patients [115]. Furthermore, we have demonstrated that the inhibition of TNF-α reduces the pro-angiogenic drive of senescent macrophages [115].

**Table 2:** Summary of evidence for inflammaging and immunosenescence in AMD: systemic vs. local.

Immune Compartment or Tissue	Cell or Tissue Type	Systemic	Local (Choroid and Retina)
Innate Immunity	Neutrophils	Transmigration of activated neutrophils from the circulation into the retina during early AMD [116].	Infiltration of LCN-2^+^ neutrophils into choroid and retina, associated with increased IFN-λ levels in the retina [117]. Elevated IL-17 levels associated with neutrophil recruitment into tissue [118].
	Monocytes–Macrophages	Higher peripheral monocyte counts [119]. Reduced monocyte phagocytic function across monocyte subtypes [73]. Increased activated monocytes associated with elevated TNF-α levels [120]. Elevated IL-17RC expression by CD14^+^ monocytes—role for IL-17 in AMD [121]. Impaired cholesterol efflux in senescent macrophages [104]. Circulating and infiltrating monocyte/macrophage SASP markers: IL-1α, IL1-β, IL-6, IL-8, IL-12, TNF-α, C3, CFB, CXCL1, TGF-β and nitric oxide [43,65].	Elevated CCR2^+^ monocytes detected in subretinal space [122]. Increased macrophage recruitment into the choroid [123]. Retinal microglia SASP markers: IL-1α, IL1-β, IL-6, IL-8, IL-12, TNF-α, C3, CFB, CXCL1, TGF-β and nitric oxide [43,65]. Macrophage production of atypical lipid species/aberrant lipid production [105]. Increased macrophage VEGF-A production (experimental models) [114].
	Mast cells	Context-dependent SASP, e.g., CXCL1 production [74,75].	Increased mast cell degranulation in the choroid of AMD patients [123].
	Natural Killer (NK) cells	The HLA-Cw*0701 allele and KIR ligand haplotype AA are associated with AMD [124]. Lower prevalence of resting NK cells in AMD patients [125].	Experimental models: in vivo depletion of IFN-γ-secreting NK cells in a mouse model leads to reduced CNV [126].
Adaptive Immunity	T cells	MHC Class I and II polymorphisms associated with the development of AMD [127,128]. Increased percentages of CD56^+^ and CD28^-^ T cell populations with increased cytotoxic function [78].	CD8^+^ T cells observed in macula of donor eyes with AMD [5]. IL-17 detected within AMD lesions [44,129]. Tissue-specific γδ T cells source of IL-17 in the outer retina [130]. Experimental model: IL-17 promotes choroidal neovascularization [131].
	B cells	Anti-retinal auto-antibodies (e.g., specific for GFAP, drusen constituents and ECM) described at all stages of AMD, suggesting presence of auto-reactive B and plasma cells. [132,133]	Limited evidence of B cell activity/presence in the choroid. Experimental models: immune complex deposition leads to microglia activation and recruitment of myeloid cells [134].
Interface- “Professional” antigen presenting cells (APCs)	Dendritic cells (DCs)	No clear evidence to suggest differences in systemic DC numbers or activity in AMD patients.	Choroidal DCs express higher levels of S100A8/9 [84,98]. Experimental model: increased levels of MHC class II^+^ DCs in a murine model of light-induced retinal degeneration [135].
Stroma, Vasculature or Peripheral Blood	Tissue (as specified)	Choroidal vasculature: Senescent changes in choroidal endothelial cells sensitize the choriocapillaris to MAC-induced endothelial dysfunction [136].	RPE: elevated IL-18, NLRP3, activated caspase-1 (evidence of inflammasome activation) [99]. RPE cells lose CD46 (regulator of complement activation) early in GA development [137]. Majority of complement MAC occurs in domains surrounding the choriocapillaris, not the RPE or retina [138]. *SPARCL1* gene enriched in choriocapillaris endothelial cells in early atrophic AMD eyes [98].
	Serum or plasma	SASP markers (detected in serum of AMD patients): IL-6, IL-8, IL-12, MCP-1, TNF-α, IL-1α, IL-1β, and IL-17, IL-22 [43,44]. Plasma: elevated levels of C3a and CRP [94,95,96].	SASP markers (detected in aqueous of AMD patients): IL-6, IL-8, IL-12, MCP-1, TNF-α, IL-1α, IL-1β, and IL-17.

Abbreviations: C-reactive protein (CRP); Extracellular matrix (ECM); Glial fibrillary acidic protein (GFAP); Killer cell immunoglobulin-like receptor (KIR); Lipocalin-2 (LCN-2); Membrane attack complex (MAC); Nod-like receptor family pyrin domain containing 3 (NLRP3); Senescence-associated secretory phenotype (SASP); SPARC-like protein 1 (SPARCL1); Tumor necrosis factor-α (TNF-α).

Studies have demonstrated that peripheral monocyte counts are higher in AMD patients than control patients [119]. Interestingly, in patients with intermediate and advanced AMD, monocyte phagocytic function was shown to be reduced across several monocyte subtypes compared to control patients [73]. Furthermore, AMD patients with a high prevalence of CNV were demonstrated to have higher levels of activated monocytes, and this was associated with an elevated expression of TNF-α [120]. Studies amongst siblings have shown that peripheral blood CD14^+^ monocytes expressing the IL-17RC receptor are elevated in those siblings with AMD compared to those without [121]. This suggests that the interaction of IL-17 with its receptor IL-17RC on CD14^+^ monocytes plays a role in AMD pathogenesis [121]. In addition, CCR2+ monocytes have been detected both in the subretinal space of patients with AMD [122], and in a mouse model, shown to promote photoreceptor degeneration.

Inflammatory monocytes in the peripheral circulation of nAMD patients also show an upregulation of CCR1 and CCR2 [139], presumably supporting the recruitment of these cells to the retina via chemokine signaling. In addition, gene expression in monocytes from AMD patients show an inflammatory expression signature compared with age-matched controls [140]. Potentially, dietary supplementation or diet pattern can affect monocyte function in AMD [141], thereby modulating the involvement of these cells in the disease [141,142].

### 4.2. Mast Cells and Context-Dependent SASP

A comprehensive gene expression atlas of retina, RPE and choroidal cell types has been generated in the last five years [123]. This provides the means to study individual populations of resident immune cells within the choroid. Interestingly, mast cell degranulation and increased macrophage recruitment into the choroid have been implicated in AMD pathogenesis [123].

Aged tissues have been shown to exhibit increased numbers of mast cells in close opposition to venular walls, similar to data from aged human skin [74,75]. Mast cells from aged tissue are a significant source of CXCL1, and this may be linked to a context-dependent SASP which ultimately facilitates enhanced vascular permeability. The oxidative stress environment of aged tissue is a key inducer of senescence. It has been speculated that mast cells adherent to ECM molecules might release increased levels of pro-inflammatory mediators [74,75]. Furthermore, tissue-resident mast cells may have a key role in the dynamics of neutrophil trafficking in aging (discussed further below).

### 4.3. Neutrophils and Their Extracellular Traps—Contributions to Immunosenescence and a Role in AMD

Neutrophils play a central role in innate immunity and are typically associated with its first wave of invading leukocytes. Several questions remain about the effect of aging on innate immunity and neutrophils in particular [143]. Experiments with aged mice have demonstrated the association of impaired neutrophil trafficking with aberrant production of systemic or local inflammatory mediators and/or reduced anti-inflammatory mechanisms [144,145,146].

A recent study investigated the effect of ageing on neutrophil diapedesis [71]. Interestingly, in the inflamed tissues of aged mice, there was an increased prevalence of neutrophils migrating with retrograde motility within endothelial cell junctions (i.e., neutrophils re-entering the vascular lumen). This was mediated by an upregulation of the chemokine CXCL1 by tissue-resident mast cells. The DARC (ACKR1)-mediated pro-inflammatory state of aged venules induced excessive ligation and desensitization of CXCR2 expressed by neutrophils, resulting in a loss of neutrophil directional motility [71]. The upregulation of mast cell-derived CXCL1 and endothelial cell DARC was shown to mediate age-related changes in the inflammatory milieu, capable of promoting neutrophils to re-enter the systemic circulation and cause remote organ injury—in this scenario, neutrophils were tracked from inflamed tissues to the lungs. DARC expression is increased in chronic inflammatory settings [147]. In light of these findings, it is interesting to note that the 15-year follow-up of the Beaver Dam Eye Study demonstrated an association between pulmonary disease and the incidence and progression of AMD [56].

Studies have demonstrated the infiltration of lipocalin-2 (LCN-2)-positive neutrophils into the choroid and retinae of early AMD patients and in murine AMD models [117]. Furthermore, increased levels of IFN-λ in the retinae of patients with early AMD were shown to trigger both neutrophil activation and LCN-2 upregulation, which, in turn, signals the transmigration of neutrophils from the circulation into the retina during early AMD [116]. IL-17 expression has also been shown to recruit neutrophils to tissues [118].

Neutrophil extracellular traps (NETs) have been described as an immune defense mechanism which are deployed by neutrophils against invading bacteria and fungi [148]. NETs incorporate a mesh of DNA tangled with granular proteins, including myeloperoxidase (MPO), elastase and cathelicidin [148]. MPO is found at high levels in the primary granules of neutrophils [143]. NETs can also trap erythrocytes and platelets, promoting vascular occlusion. It has been demonstrated that NETs are present during phases of vascular remodeling (when neutrophil levels are elevated, e.g., in diabetic retinopathy) [149]. NETs have been shown to target senescent vascular endothelial cells during the late-stage sterile inflammation that accompanies vascular remodeling in proliferative vascular retinopathies [150]. NETs eliminated diseased senescent vasculature by promoting apoptosis of endothelial cells. Inhibiting the neutrophil receptor CXCR2 impaired the clearance of senescent cells [150]. Crespo-Garcia et al. demonstrated that pathological vasculature engages p16^INK4A^ and BCL-xL (expressed by senescent cells) [151]. Furthermore, clearance of p16^INK4A^-expressing cells suppressed pathological angiogenesis and a BCL-xL inhibitor also suppressed neovascularization [151]. This raises prospects for a potential mechanism of treatment—that is, senolytic drugs that target senescent cells—in age-related eye diseases, including AMD.

### 4.4. Natural Killer Cells and AMD

Natural Killer (NK) cells have a major role in clearing or ‘killing’ virally infected cells or tumorigenic cells that manifest with reduced MHC class I ‘self’ antigens. NK cells were first postulated to have a role in the pathogenesis of AMD when our group undertook a genotyping study demonstrating that the HLA-Cw*0701 allele and killer cell immunoglobulin-like receptor (KIR) ligand haplotype AA are associated with AMD [124]. In a more recent gene expression study, there was a lower prevalence of resting NK cells in AMD patients compared to control patients. However, the C1S, ADM and 1ER5L genes were shown to correlate positively with NK cell activation, in addition to AMD progression [125].

A study has also demonstrated that in vivo depletion of interferon-γ-secreting NK cells in a mouse model leads to reduced CNV [126]. Interestingly, the inhibition of NK cell activity (in addition to CD8^+^ T cells) is one of the mechanisms through which senescent cells evade immune clearance. Senescent cells were shown to do this via expression of HLA-E [76]. Furthermore, senescent cells also upregulate the immune recognition cell surface receptor natural killer group 2D (NKG2D), which is not expressed by non-senescent cells [77]. This may facilitate the design of new immunotherapeutic strategies that focus on eliminating these NKG2D-expressing senescent cells [77].

## 5. Immunosenescence and the Adaptive Immune System

The immune suppressive functions of the RPE become compromised in AMD, and retina-infiltrating T cells [5,152] and auto-antibodies against retinal antigens have been identified in AMD patients at all stages [132,133]. This would suggest that the adaptive immunity plays a role in the pathogenesis of AMD.

### 5.1. Anti-Retinal Autoantibodies—Evidence of Senescent B Cells in AMD?

The oxidation of lipids and proteins has been suggested to generate new antigens to which the immune system does not display tolerance [153]. Autoantibodies specific for retinal and/or RPE antigens could be induced secondarily to tissue damage occurring during AMD. An example of this is the oxidation of docosahexaenoate (DHA)-containing lipids, which generates carboxyethylpyrrole (CEP) protein derivatives. These CEP-containing proteins have been detected at higher levels in drusen and blood from AMD patients compared to healthy donors [154,155].

Autoreactive T cells recognizing these neoantigens, or damage-associated molecular patterns (DAMPs), could theoretically provide help to autoreactive B cells that will secrete autoantibodies recognizing a large spectrum of retinal and RPE antigens modified by oxidation by-products [156]. Autoreactive T- and B-cell interactions could therefore result in secreted auto-antibodies recognizing modified retinal/RPE antigens [29,153].

Several retinal autoantibodies have been described in AMD patients that partly react with unknown retinal proteins of varying molecular weight (e.g., a neurofilament protein in photoreceptor outer segments) [157,158]. Anti-retinal antibodies specific for glial fibrillary acidic protein (GFAP), expressed by astrocytes and Müller cells in the retina, were detected at elevated levels compared to age-matched control patients and those with other retinal diseases [159]. Antibodies specific for approximately 30 antigens have been detected at elevated levels in the serum of patients with either atrophic or neovascular AMD compared to control patients [160,161]. This includes elevated serum levels of autoantibodies specific for drusen constituents and extracellular matrix in Bruch’s membrane [160,161]. Murine studies have demonstrated that the deposition of immune complexes (antibody linked to antigen) in the retina resulted in a localized inflammatory response with microglia activation and recruitment of myeloid cells. This suggests that immune complexes may contribute to AMD pathogenesis through the interaction of IgG with FcγRs [134].

Despite evidence of elevated systemic levels of anti-retinal autoantibodies in AMD patients relative to age-matched controls, there is no firm evidence demonstrating the association of senescent B cells with the induction or progression of AMD.

### 5.2. T-Cell-Based Immunosenescence and Implications for AMD—IL-17 and γδ T Cells

In 2005, we demonstrated that specific MHC class I and II polymorphisms were associated with the development of AMD, which suggested a role for T cell responses in pathogenesis [127,128]. At the mRNA level, it has been demonstrated that genes specific for T cells (e.g., CD3, CD8 and CD205/DEC-205), antigen presentation (e.g., β2 microglobulin and H2 molecules), T cell chemotaxis (e.g., CXCL9-11 and CCL5) and adhesion molecules were upregulated in the retina and RPE/choroid with increasing age [82,83].

Some of the hallmarks of immunosenescence relate to overall numbers and proportions affecting the T lymphocyte compartment of the adaptive immune system. This includes a reduction in peripheral lymphocyte numbers and increased frequencies of memory CD4 and CD8 T cells [29,31]. This is accompanied by the infiltration and activation of myeloid cells in tissues [29,31]. Within the CD4 helper T cell compartment, there is a relative increase in frequency of Th17 cells [81] but reduced inducibility and stability of regulatory T cells (Tregs). This is important as Tregs are a population of CD4+ T function to actively suppress antigen-specific CD4^+^ and CD8^+^ T cell activation and differentiation via cell-contact, IL-10 and/or TGF-β secretion [162]. Tregs co-express the IL-2 Receptor (CD25) at high levels (CD4^+^CD25^hi^), and naturally occurring Tregs express the transcription factor *FoxP3*, used as a marker for these cells [163]. Tregs can also be induced by IL-10 (Treg1 cells).

Interleukin-17 (IL-17) is a potent pro-inflammatory cytokine that has been implicated in a range of age-related autoimmune and inflammatory diseases [164]. For example, IL-17 expression leads to the recruitment of monocytes [165], activation of tissue-based macrophages and enhancement of their phagocytic capacity [166,167]. Elevated levels of IL-17 have been detected in the serum of AMD patients compared to the levels observed in age-matched controls [129]. mRNA and protein levels of the IL-17 receptor subunit C have been detected at elevated levels in macular tissues of AMD patients [121]. This suggests that IL-17-producing cells are involved in several pathological processes observed in AMD. IL-17 has been demonstrated to promote choroidal neovascularization in a mouse model by the growth of vessels in the sub-retinal space [131]. This has either been via enhancement of endothelial cell growth or by inducing production of VEGF by other cell types [168,169].

The presence of retina-infiltrating memory- and IL-17-producing T cells have long been speculated upon in AMD [153], associated with reduced numbers of Tregs [31]. It has also been speculated that either CD4^+^ T helper cells or CD8^+^ T cytotoxic cells have an IL-17-producing population which have a significant role in AMD pathogenesis [170]. CD8+ T cells have been observed by fluorescence microscopy in the macular choroid of frozen sections of eyes from AMD patients [5]. The tissue sections from AMD patients also contained drusen deposits.

Senescent T cells have been associated with a unique array of pro-inflammatory cytokines [171]. Furthermore, these cells display a loss of costimulatory molecules CD28, CD27 and CD40L [172,173]. Within the peripheral blood memory T cell compartment, it was demonstrated that there are elevated percentages of CD56^+^ and CD28^-^ populations in AMD patients relative to age-matched controls [78]. Furthermore, increased populations of CD56^+^ and CD28^-^ memory T cells displayed increased cytotoxic function, and this was associated with a significantly greater risk of developing AMD [78]. This risk is further elevated in the presence of the *CFH SNP* rs1061170 (Y402H) [78]. The increase in interferon-γ production through aging also correlates with an expanded CD8^+^CD28^-^CD57^+^ T cell subpopulation [79].

γδ T cells have been demonstrated to produce a large amount of IL-17 in tissue-specific defense and autoimmune responses [174]. γδ T cells are named for their unique T cell receptor (TCR)- a dimer of the γ and δ chains (in comparison to the commonly studied αβ T cells). γδ T cells respond to antigens with lower specificity but faster kinetics, in addition to producing effector cytokines such as IL-17 and IFN-γ independent of TCR ligation [80,174,175]. IL-17 produced by γδ T cells and innate lymphoid cells have been demonstrated to promote experimental intraocular neovascularization using the laser-induced CNV murine model [131]. In a more recent study, it was concluded that tissue-specific γδ T cells were the main source of IL-17 in the outer retina [130]. γδ T cells have been discovered in the sub-RPE space during age-dependent RPE degeneration [130].

Interestingly, IL-22 and IL-17, both of which are produced primarily by the Th17 compartment of T cells, were detected at higher levels in the sera of AMD patients, in addition to IL-17 detected within AMD lesions [44,129].

## 6. Dendritic Cells at the Interface of Innate and Adaptive Immunity in AMD

Dendritic cells (DCs) are professional antigen-presenting cells that also serve as a link between the innate and adaptive immune systems. In addition to resident and infiltrating (monocyte-derived) macrophages, A. Voigt et al. recently identified a population of DCs based on their choroidal transcriptomes [100]. Increased levels of MHC class II^+^ DCs, together with T cells, have been demonstrated in murine models of light-induced retinal degeneration [135].

Choroidal DCs, in addition to choroidal resident macrophages, have been shown to express higher levels of S100A8/A9 in AMD relative to control eyes [84,100]. S100A8/A9 are calcium-binding proteins that have pro-inflammatory properties and can promote leukocyte recruitment [176].

## 7. The Immunoregulatory Role of the Complement System at the Interface of Innate and Adaptive Immunity

There is a complex interplay between the innate and adaptive immune systems in the progression of AMD, and this is associated with the complement system. Complement activation involves both humoral and cellular immune components, including the recruitment and activation of leukocytes, secretion of inflammatory cytokines and breakdown of the blood–retina barrier, therefore contributing to chronic inflammation and tissue damage [19]. Several studies have shown that elevated levels of complement activation fragments are independently associated with AMD [177,178]. Additionally, complement activation has been demonstrated to be associated with the stage of AMD [179]. Elevated serum levels of C5a in AMD patients stimulate the production of IL-22 and IL-17 by T cells [129].

An immunoregulatory role of the complement pathway has also been described, possibly bridging innate and adaptive immunity [19]. Complement dysregulation in the choroid is of significant interest in AMD, and choroidal cell types express more complement inhibitors than the overlying retina or RPE [138]. These regulators include CD46, CD55, CD59, CD93 and Complement factor H [138]. CD46 acts as a regulator of complement expression: it is a cofactor for complement factor I, protecting autologous cells against complement-mediated injury by cleaving C3b and C4b deposited on the cell surface [180]. Co-ligation of the T-cell receptor (TCR) and CD46 on CD4^+^ T cells induced a Treg-specific phenotype in the presence of excess IL-2 [181]. IL-10-producing cells were shown to both proliferate and suppress the activation of bystander T cells [182].

RPE cells have been demonstrated to lose CD46 expression very early in the development of GA, prior to any morphological change of RPE [137]. The loss of CD46 expression therefore makes RPE cells vulnerable to complement-mediated damage. CD46 expression by RPE also has a role in the adhesion of this monolayer to its basement membrane via association with beta-1 integrin [183]. Interestingly, the reduced expression of CD46 by RPE/choroid results in drusen-type pathology in mice [184]. Additionally, CD46 expression protects against laser-induced CNV in mice [185].

## 8. Barriers to Overcome—Alternative Therapies in AMD and the Future Role of Complement Inhibition

### 8.1. Choroidal Vasculature, Senescence and Complement-Mediated Damage

AMD is characterized by vascular degeneration, with choroidal endothelial cells degenerating very early in the disease course [186,187]. Histological and imaging studies have demonstrated that the degeneration of the superficial choriocapillaris is the first observable event in the pathogenesis of AMD, under an intact, confluent RPE monolayer [13,188]. Aging choroidal endothelial cells in rhesus monkeys have been shown to exhibit high levels of SA-β-Gal and aberrant cytoskeletal activity [136]. These senescent changes in choroidal endothelial cells sensitize the choriocapillaris to MAC-induced endothelial dysfunction [136]. The senescence of retinal vascular endothelial cells has also been observed in the aging retina [89]. Aging choroidal endothelial cells in rhesus monkeys have been shown to exhibit high levels of SA-β-Gal and aberrant cytoskeletal activity [136]. Therefore, the senescence of either type of vascular endothelial cell (choroidal or retinal), and consequently, vascular dysfunction plays a potentially important role in CNV, including in AMD.

The degeneration of choriocapillaris endothelial cells is therefore, in part, due to MAC injury, and choroidal endothelial cells are susceptible to MAC-mediated lysis [138]. The majority of complement MAC occurs in domains surrounding the choriocapillaris, not the RPE or retina [138]. Maintaining an intact choriocapillaris by preventing MAC-mediated lysis is therefore important for macular health.

SPARC-like protein 1 (SPARCL1) is a secreted glycoprotein that is expressed in response to cell injury [189]. In addition, this matricellular protein promotes cellular quiescence and stimulates the detachment of cells from basal lamina through focal adhesion disassembly [190,191]. Similarly, the related protein SPARC (described in the context of macrophages above) is uniquely upregulated in the setting of cell injury [189]. It has been demonstrated that the *SPARCL1* gene was enriched in choriocapillaris endothelial cells in early atrophic AMD donor eyes [100]. SPARCL1 expression results in decreased endothelial cell adhesion to the basement membrane and increases the spreading of endothelial cells in vitro [192]. In human choroidal sections, SPARCL1 localizes to choroidal endothelial cells (including the choriocapillaris) in AMD across the entire vascular tree [100]. More specifically, SPARCL1 was shown to be expressed at the protein level in a perivascular pattern around choroidal endothelial cells, including the choriocapillaris. This is relevant because, as described above, choroidal endothelial cell loss can be observed early in AMD [186,187]. SPARCL1 is additionally cleaved by ADAMST4 [193], generating a shorter fragment that is associated with neovascularization [194].

Zeng et al. sought to identify strategies to protect choroidal endothelial cells against MAC-mediated lysis. The group demonstrated that imidazole compounds (econazole nitrate and miconazole nitrate) reduced the lysis of choroidal endothelial cells treated with complement-intact serum [195]. This study showed that small molecules can protect choroidal endothelial cells from MAC-induced cell death [195].

### 8.2. Immunomodulatory Therapy/Immunosuppression and Current Barriers

As both the innate and adaptive components of the immune system have been implicated in inflammaging and AMD, it has been speculated that immunomodulatory therapy might reduce the risk of AMD progression. Caloric restriction in humans has been demonstrated to lower inflammatory markers in the blood without risking an increase in infections [196]. It has been proposed that caloric restriction mimetics that reduce inflammation can possibly target chronic diseases. However, this may not be practical in all patients and is non-specific.

Intraocular corticosteroid therapy in AMD has shown little efficacy [197,198]. However, a study of concomitant systemic immunosuppressive therapy did show initial promise in reducing the frequency of intravitreal anti-VEGF injections in AMD [199]. Rapamycin is commonly used as part of immunosuppression regimens (e.g., post-transplantation). Systemic immunosuppression with rapamycin has been demonstrated to inhibit CNV in animal models [200]. Interestingly, the nutrient-sensing mTOR signaling pathway has a role in cellular senescence [201], and the inhibition of mTOR signaling has been shown to repress autophagy in animal models [202]. Treatment with low-dose rapamycin, which is a specific inhibitor of mTORC1 and thus mTOR signaling, has been shown to inhibit RPE senescence in vitro, with a decreased percentage of senescent cells and reduced SA-β-Gal activity [201]. mTOR inhibition by rapamycin also prevented photoreceptor degeneration induced by RPE stressor exposure [203].

Sandhu et al. demonstrated that systemic immunosuppressive or immunomodulatory therapy did not affect the risk of onset or progression of AMD: a retrospective cohort study was carried out on post-kidney transplantation patients who were on at least one systemic immunosuppressive therapy vs. age-matched chronic kidney disease (CKD)ban stage IV or V patients on no treatment [204]. Mycophenolate mofetil, however, was shown to confer some degree of protection against the conversion of dry to wet AMD [204]. This suggests that the modulation of the immune response may reduce the risk of disease progression. However, the main issues with immunomodulatory or immunosuppressive therapy in AMD discussed here are that they do not target a specific component of the immune system and could also pre-dispose patients to an increased risk of infections.

### 8.3. Targeting Senescence and SASP

Alternative therapeutic strategies in AMD can include the selective clearance of senescent cells, including the use of small molecules to target senescent cells that are resistant to apoptosis, or blocking/reducing SASP-associated inflammatory mediators [40,50,205].

It has been reported that senolytic effects are observed when first-generation senolytic drugs such as dasatinib (a pan-tyrosine kinase inhibitor) or quercetin (a naturally occurring flavonoid) are used, and the senolytic effect is enhanced when dasatinib and quercetin are used in combination compared to either drug alone [205]. For example, these drugs have been evaluated in clinical trials with patients with diabetic kidney disease [206], with a reduction in p16^INK4A^- and p21^CIP-1^-expressing adipocytes and circulating SASP factors. A small molecule, UBX1967, which targets the anti-apoptotic protein B cell lymphoma-xL (BCL-xL), has been shown to target senescent cells as a potential treatment for neovascular retinal disease [151]. A current phase 2, prospective, multicenter randomized study is assessing the small molecule UBX1325 (a BCL-xL inhibitor) in the treatment for neovascular AMD [207]. It has been suggested that senolytic drugs will have optimal effects on inhibiting systemic immunosenescence and SASP after systemic administration [205].

It has also been suggested that senolytic therapy may compromise the beneficial effects of senescence, including its anti-tumor effects. An alternative strategy to therapy would be to reduce or block SASP associated with immunosenescence. A significant number of pro-inflammatory cytokines associated with SASP have signaling pathways that ultimately activate the NF-κB pathway and are therefore potentially responsive to modulation. For example, the use of neutralizing antibodies against IL-1α or its receptor can reduce NF-κB transcriptional activity as IL-1α/ IL-1α receptor signal transduction is upstream of NF-κB [208]. In addition, approved drugs such as tocilizumab or siltuximab, which target IL-6/IL-6 signal transduction, could also target senescence. As described earlier, elevated levels of IL-17 have been detected in the serum of AMD patients compared to the levels observed in age-matched controls [129]. Interestingly, humanized anti-IL-17 antibodies, secukinumab and ixekizumab, have been investigated in phase 2 clinical trials for the treatment of rheumatoid arthritis [209]. Blocking chemokine signaling may also serve as a therapeutic strategy in atrophic and nAMD via a reduction in monocyte recruitment to the affected retina [210].

## 9. Conclusions—The Prospect of Combination/Adjunct Therapy for AMD

The clear role of the complement system in causing early damage in AMD (e.g., the role of MAC in choriocapillaris endothelial cell apoptosis) and its role in activating both innate and adaptive immunity mean that adjunct/combination therapy may need to be considered. Focusing solely on factor H and/or factor I supplementation or inhibiting complement components may not be adequate in light of the role of systemic immunosenescence and inflammaging in AMD. Drugs that have multiple targets may need to be a strategy. An example of this would be the senolytic drugs described earlier, or drugs such as rapamycin, which, at low dose, inhibits mTOR signaling, can block SASP by reducing the expression of surface/membrane-bound IL-1α [211], and can inhibit senescence. As most patients do not progress to develop the advanced forms of AMD, screening patients for combination therapy would be as important as the therapeutic strategy itself.

## Data Availability

Data sharing not applicable.
